# Bonsall’s Address

**Published:** 1858-12

**Authors:** C. Bonsall


### ADDRESS

Before the Cincinnati Association of Dental Surgeons, formed September 3d, 1858, by its first President,

DR. C. BONSALL.

*Fellow Members of the Cincinnati Dental Association :*

Gentlemen : Having been requested by you to prepare an address for our Association, at its first regular meeting after organization, I have thought a comparison would be profitable of the condition of the profession, and the facilities which are enjoyed by those of the present day, who wish to commence Dental practice reputably, compared with those which were attainable in former years, and which improvements are mainly to be attributed to to the influence exercised directly and indirectly by the associating together of liberal minded Dentists for similar purposes to those which have induced you to unite in this Society.

The Society known as the American Society of Dental Surgeons, having been the first combination of Dentists for this purpose with which I am acquainted, and feeling conscious that the fact of having been elected as one of its members at an early day, was one of the means of liberalizing my own views, from the anxiety it produced in me, that I might become worthy to meet with such bright ornaments to the profession, as many of the earlier members of that society were, I have been induced to turn to the early records of the society, which happen to be in my possession, and shall copy here the remarks made before the few honorable dentists who had come together from different States of the Union, in the City of New York on the 18th day of August, 1840, for the purpose of forming a Society for mutual improvement, and to endeavor to ennoble the profession ; by that ornament to the profession the lamented Hayden.

In the first place I will mention that the initiatory steps to this meeting had been taken by Dr. Horace H. Hayden of Baltimore, having sent invitations to some thirty-five or forty of the best known members of the profession from almost all parts of the Union, which resulted in the coming together of fifteen to twenty of those who had been invited, at that time, at the American Hotel in New York.

Before adopting a Constitution, Dr. Hayden took the opportunity of making the following statement:

"The formation of a National Society of Dentists, he remarked had been long a favorite project with himself, and he was happy to say, that he had never found himself wholly alone in this desire. As long ago as the year 1817, he had consulted with the late Dr. Edward Hudson, of Philadelphia, who was favorably inclined to such an association.

Other gentlemen of the profession, however, were not inclined to make an effort in the business, although gentlemen of the Medical Profession, to their honor be it spoken, uniformly approved of the plan.

The prospect of succeeding, however, appearing rather unfavorable, he had mostly relinquished the plan until, finding that his son manifested a desire to pursue the same profession, and contemplating a tour to the North, in 1829, he resolved to make another effort amongst his professional friends and acquaintances, and in this instance, he met with much more encouragement; but not sufficient, however, to justify a belief that the plan would succeed until the year 1838 when in a to tour the North he made the last attempt.

The speaker adverted, in the next place,, to the condition of the dental art, at the time when he entered upon its duties forty-three years ago. *Then,* said he, the name of dentist was a reproach and a bye-word. But viewing it as a subject susceptible of great and beneficial improvements by well written essays or works, and not knowing at the time whether any such had ever been published, he applied to the elder Mr. Greenwood, of New York, then dentist to Gen. Washington, who informed him, that he knew of but one work that had ever been published, and that was Dr. John Hunter's work on the natural history of the teeth, which he procured. Soon after he obtained possession of some few other works, both in French and English.

From that time he has seen the science assuming more and more importance in the public eye, whilst men of learning, worth, and genius, have been added from year to year, to the rank of its Professors.

At the present period, continued the speaker, renewed efforts are making in England, France, and Scotland, to place our profession on still higher ground than it has yet attained; and shall we of these United States of America, remain inactive in this grand endeavor.

For myself, said he, I can not brook defeat in this favorite undertaking. I prefer rather to make renewed and redoubled efforts to secure success. If, like the Jews, we are scattered and despised, may we not, like that persecuted people, at least hope for a day of restoration to our promised land. There are many obstacles, indeed, still to overcome. A new race of Canaanites must be expelled from our borders. Many errors must be exploded, and much ignorance must be dispelled, before the light of truth will beam clearly upon our path; but with diligence, zeal, and perseverance, we are certain of ultimate success.

Let us, therefore, go forward in the good cause, unintimidated by the skepticism of the faithless, the fear of the timid, or the apathy of the selfish.

If there are *some* who prefer to plod onward in the path of private enterprise, *let us* unite our efforts in one great social endeavor, to elevate our profession from the degraded condition to which it has sunk, and in which it must ever remain, until the high-minded and well-educated among the practitioners shall unitedly rise and shake themselves from the dust."

Dr. Hayden then called for the reading of the Constitution which had been prepared by a committee; the first article of which was as follows:

"The objects of this Society are to promote union and harmony among respectable and well-informed Dental Surgeons; to advance the science of free communication and interchange of sentiments, either written or verbal, between members of the Society,—both in this and other countries; in fine, to give character and respectability to the profession, by establishing a line of distinction between the truly meritorious and skillful, and such as riot on the ill-gotten fruit of unblushing impudence and empiricism."

The Constitution, then adopted, was signed by fifteen members.

To show the feeling and and interest felt by some of those present at this first meeting, I will quote a few words more from some remarks made by another of the bright ornaments of the profession, Dr. Eleazar Parmly, of New York: he said, "May not a noble pride be felt by our children, and even our children's children, when they read that their ancestors were the little band who met, from distant cities, to rescue a noble science from the degradation to which it had been reduced by ignorance, selfishness, and quackery; and will it be no source of pleasing recollection to ourselves, that on such a day of such a year, we met each other, in this place, and signed a document which declared us members of a profession which afforded ample scope for all the moral, intellectual and physical energies of our nature to be engaged for the general good of our fellow men."

This Society—having broken the ice, as it were, by showing the profession what might be gained by social and professional intercourse, State and other local Societies very soon followed in its footsteps, and, also, having been the means of starting the first periodical devoted to Dental Science, the "American Journal,"—continued during a period of sixteen years to exert an excellent influence over the profession; and finally gave place to the present "National Dental Convention," with the character and usefulness of which, you are all acquainted.

In our own city, a Society was formed, in 1844, composed of most of the Cincinnati dentists of the time, and continued its meetings, for some time, with benefit to the members; but finally dissolved, owing to little jealousies very natural to the then situation of the profession, which had just recently emerged from a condition in which no two dentists, hardly in any city, were on pleasant social terms. There was one lasting benefit, however, which grew out of the meeting of Cincinnati Dentists together, during the continuation of the Society, which was, the call which enamated from them to the dentists of the Mississippi Valley generally, and which eventuated in the formation, in August, 1844, of the "Mississippi Valley Association of Dental Surgeons," which, I am satisfied, has been of very great advantage to many members of the profession, scattered over the West and Southwest. But of this Society, also, I need not speak, as you are all acquainted with it.

At the present time, there are a large number of dental associations, over the United States (and recently, one or two in England) which are constantly exerting a useful influence over the younger members of the profession particularly, and even the oldest and most experienced dentist can hardly attend a meeting of any one of these Societies, without receiving some useful hints, in addition to which the social communion and acquaintance consequent upon the meetings, induces an improved manner of speaking of each other's operations, which serves to place dental practitioners, as a class, on a much higher level than they formerly were, in the estimation of the community generally. In addition to the advantage enjoyed by dentists of the present time from societies, there are others which were not known twenty years ago, in the opportunity given by the Dental Colleges; and also from periodicals devoted almost exclusively to dental science, of which we then had none, and now we have six or eight, which are well established.

With some additional care on the part of respectable practitioners in taking students, viz: declining to take any one as a student unless he has the education and manners of a gentleman,—there will be, in a few more years, a still further improvement in our operations, and also in the estimation in which the Dentist will be held by others. That this advance, in character and estimation, may be deserved, is the ardent wish of your fellow member.

